# Chaotic Ensemble of Online Recurrent Extreme Learning Machine for Temperature Prediction of Control Moment Gyroscopes

**DOI:** 10.3390/s20174786

**Published:** 2020-08-25

**Authors:** Luhang Liu, Qiang Zhang, Dazhong Wei, Gang Li, Hao Wu, Zhipeng Wang, Baozhu Guo, Jiyang Zhang

**Affiliations:** 1 China Aerospace Academy of Systems Science and Engineering, Beijing 100037, China; liuluhang@sina.com; 2 Beijing Institute of Control Engineering, Beijing 100094, China; zhang007qiang@163.com (Q.Z.); dzzhong99@aliyun.com (D.W.); liganghit@163.com (G.L.); 2858666@163.com (H.W.); zjywsy@vip.sina.com (J.Z.); 3 State Key Lab of Rail Traffic Control & Safety, Beijing Jiaotong University, Beijing 100044, China; zpwang@bjtu.edu.cn

**Keywords:** control moment gyroscope, temperature prediction, online-recurrent extreme learning machine

## Abstract

Control moment gyroscopes (CMG) are crucial components in spacecrafts. Since the anomaly of bearing temperature of the CMG shows apparent correlation with nearly all critical fault modes, temperature prediction is of great importance for health management of CMGs. However, due to the complicity of thermal environment on orbit, the temperature signal of the CMG has strong intrinsic nonlinearity and chaotic characteristics. Therefore, it is crucial to study temperature prediction under the framework of chaos time series theory. There are also several other challenges including poor data quality, large individual differences and difficulty in processing streaming data. To overcome these issues, we propose a new method named Chaotic Ensemble of Online Recurrent Extreme Learning Machine (CE-ORELM) for temperature prediction of control moment gyroscopes. By means of the CE-ORELM model, this proposed method is capable of dynamic prediction of temperature. The performance of the method was tested by real temperature data acquired from actual CMGs. Experimental results show that this method has high prediction accuracy and strong adaptability to the on-orbital temperature data with sudden variations. These superiorities indicate that the proposed method can be used for temperature prediction of control moment gyroscopes.

## 1. Introduction

Health management of attitude control systems of spacecrafts is a promising research direction at present. Recently, control moment gyroscopes (CMGs) have become the actuator of choice due to their high torque amplification capability and play essential roles in the operation of spacecrafts. CMGs are capable of producing significant torques and can handle large quantities of momentum over long periods of time. Consequently, CMGs are preferred in precision pointing applications and in momentum management of large, long-duration spacecrafts. However, owing to the short period of applications, condition monitoring and health management of CMGs is less studied. Therefore, it is essential to study this issue.

A CMG is comprised of a rapidly spinning rotor mounted on one or two gimbals, and is accordingly called a single gimbal CMG (SGCMG) or a double gimbal CMG. Due to the limitations of weight, space and energy cost, there are only a few signals (rotating speed, temperature, electric current and so on) gathered from the on-orbit satellites. Among those signals, the temperature signal shows apparent correlation with nearly all of the critical fault modes such as bearing failure, rotor jam and frame jam. Therefore, the temperature signal is an important indicator to identify whether the CMG is working well. However, considering the delay of transmission and processing, health assessment based on the monitoring of temperature signal has a certain hysteresis. Therefore, it is essential to study temperature prediction so as to assess the CMG’s health state and detect weak failures as early as possible.

Currently, a number of methods are proposed for monitoring machine temperatures [1,2,3,4]. Deng X. et al. established a mathematical model of the thermal characteristics of a spindle bearing system [5]. Bing C. et al. designed a digital temperature-based temperature alarm system [6]. Ma W. et al. used statistical methods based on historical data to study temperature prediction [7]. In recent years, the rapid development of machine learning algorithms has brought new possibilities for temperature prediction. For instance, Luo et al. proposed a long short term memory-based approach to forecast the temperature trend [8,9]. In addition, many other methods based on temperature data have also been proposed [10,11].

Considering the universal presence of chaotic phenomena and the complex environments of spacecrafts, the temperature signal also has strong intrinsic nonlinearity and chaotic characteristics. Therefore, it is crucial to study temperature prediction under the framework of chaos time series theory.

As a typical feedforward neural network, online sequential extreme learning machine (OS-ELM) is a good choice to deal with sequence data prediction [12]. Zhang et al. used an OS-ELM-based model to realize the real-time prediction of solar radiation [13]. Yu et al. proposed an improved OS-ELM method and achieved good results in the online ship rolling prediction [14]. Park et al. proposed an improved OS-ELM model called online recurrent extreme learning machine (OR-ELM). Experimental results showed that the model can not only obtain high prediction accuracy, but it also has remarkable adaptability to mutation data [15]. However, those individual algorithms have variations in different trials of simulations and relatively poor stabilities [16]. To overcome this weakness, on the basis of chaos time series theory, we propose an integrated structure to ensemble a number of OR-ELMs as a whole, called Chaotic Ensemble of Online Recurrent Extreme Learning Machine (CE-ORELM). This structure attempts to improve the stability and accuracy by synthetic analysis according to results of several individual OR-ELMs.

In this paper, a CE-ORELM-based method is proposed for temperature prediction of control moment gyroscopes. The structure of this paper is arranged as follows: Section 2 describes the challenges of the CMG temperature prediction problem; Section 3 introduces our prediction framework for the temperature of CMG; Section 4 introduces the experiments details; in Section 5 we illustrate experimental results and make some discussion about the result; Section 6 concludes the work of this paper.

## 2. Challenges of Control Moment Gyroscope Temperature Prediction

As aforementioned, since the inherent relationships and operation environments of control moment gyroscopes are complex and rough, there are many challenges during temperature prediction.

### 2.1. Chaotic Characteristics of Raw Signals

According to chaos theory, chaos phenomena is ubiquitous. Since the temperature signal of CMG is a typical chaos time series, strong intrinsic nonlinearity and chaotic characteristics can be found and have critical influence on the prediction. Therefore, we have to study temperature prediction under the framework of chaos time series theory.

### 2.2. Poor Quality of Training Data

The monitoring signals are transmitted from spacecrafts to the ground. Due to the limitations of transmission modes and hardware resources, the sampling frequency of data obtained by the ground is greatly reduced. The problem of missing data also occurs from time to time. In addition, the data delay and noise interference in the transmission process directly lead to further reducing of data quality.

### 2.3. Large Individual Differences

The running conditions and environments of CMGs are quite different, and the distributions of temperatures are also related to these factors. It is almost impossible to train one model which can be universally applied to all CMGs. Even if a model is trained that performs well in offline prediction, its performance may greatly reduce in practice.

### 2.4. Streaming Data Processing

The temperature data are transmitted to the computing platform in the form of network byte stream. The collection, preprocessing and model training method of the streaming data are different from the traditional offline training approach. The streaming data is usually unstable, which requires the algorithm to have the ability of continuous learning and timely adaptation to these changeable features.

Currently, there is no existing model which can solve all aforementioned challenges. Concerning those issues, we introduce a new efficient and accurate prediction framework which is also verified by using actual data collected from CMGs in service.

## 3. Methods

### 3.1. Phase-Space Reconstruction of Chaotic Time Series

The dynamic phase-space reconstruction theory is an important method to study in dynamic systems [17]. It extends a one-dimensional chaotic time series to a high-dimensional phase space, which can better describe the dynamic morphology of the system. For time series *x*(*t*), *t* = 1, 2, 3, …, *N*, the system embedding dimension (*m*) is introduced to construct an m-dimensional phase space, in which *x*(*t*) can be expressed as:

(1) X(t)=(x(t),x(t−1),⋯,x(t−(m−1))).

According to Takens’ embedding theory [18], as long as the dimension satisfies:

(2) m≥2D+1,

where *D* is the dimension of the system attractor, then the reconstructed system is equivalent to the original dynamics system.

Therefore, the determination of the system embedding dimension m is the key issue for phase space reconstruction. Therefore, we need to calculate the system attractor dimension *D*.

In general, the correlation dimension is calculated as Grassberger and Procaccia proposed an algorithm for calculating the correlation dimension [19], also known as the G–P algorithm, which is widely used. The steps of the algorithm are as follows:

(1) Calculation of correlation integral:

Define *N* as the number of vectors in reconstructed phase space, and the correlation integral *C_m_*(*r*) is defined as

(3) $$C_{m}\left(r\right)=\frac{2}{N\left(N-1\right)}\sum_{i=1}^{N}\sum_{j=i+1}^{N}H\left(r-\left|X_{i}-X_{j}\right|\right),$$

where, *H* is the Heaviside function.

(2) A cluster of ln *C_m_*(*r*)-ln(*r*) curves are plotted through increasing *m*. Then the least squares method is used to make linear regression in the curve’s approximately linear part, to obtain the estimated value of correlation dimension *D*.

The correlation dimension *D* is defined as

(4) $$D=\underset{r\to 0}{\lim}\frac{\ln\left(C_{m}\left(r\right)\right)}{\ln\left(r\right)}.$$

### 3.2. OR-ELM Theory

As a widely used solution to the online time-series prediction problem, online sequential extreme learning machine (OS-ELM) can quickly track new sequence patterns and performs better than other online learning solutions in most cases [12]. Due to the introduction of an incremental learning algorithm, OS-ELM can update the model parameters sample-by-sample. When new samples are added, it is not necessary to recalculate all the previous data, but to conduct incremental learning on the new sample based on the previous model.

**Figure 1:**
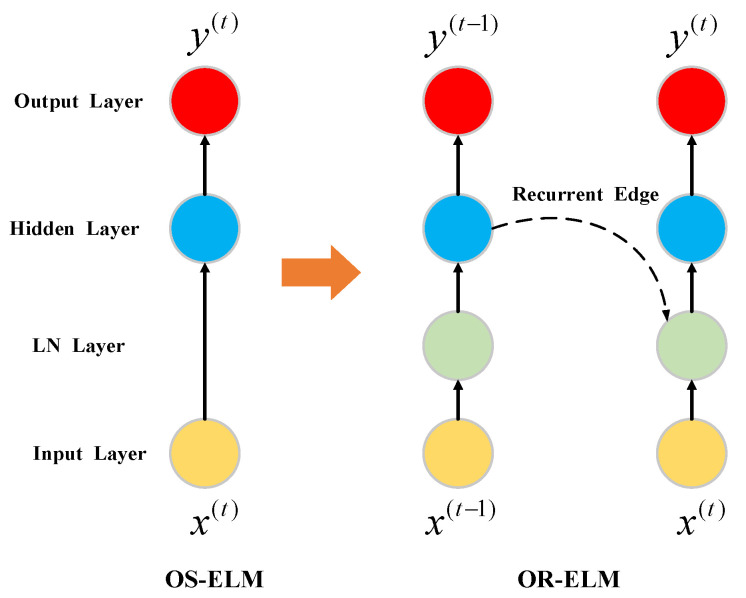
A simple online sequential extreme learning machine (OS-ELM) model and a simple online recurrent extreme learning machine (OR-ELM) model.

Park et al. [15] proposed an improved OS-ELM, called online recurrent extreme learning machine (OR-ELM), which shows better performance on time-series prediction tasks than other traditional online learning methods such as hierarchical temporal memory (HTM) and online long short-term memory (online LSTM). The OR-ELM algorithm adds an LN layer to the basic OS-ELM and constructs a recurrent neural network as its main framework. The weights of input layer and hidden layer are updated by two ELM-auto-encoders (ELM-AE) [16], the first being ELM-AE for input weight (ELM-AE-IW) and the second ELM-AE for hidden weight (ELM-AE-HW). The learning process of OR-ELM is mainly divided into two stages. The first part is the initialization stage, in which the input weight, output weight and parameter matrix are initialized. The second part is the online sequential learning phase, in which a new chunk of samples is used to update the input weight, output weight and hidden weight. Figure 1 shows the difference between a simple OS-ELM model and the corresponding improved OR-ELM model.

1. Initialization stage:

For any online prediction model, there is no training data available in the initialization process, so the algorithm adopts the method to complete the initialization of the output weight $\beta_{0}$:

(5) $$\beta_{0}=0,\;\;\;\;P_{0}={\left(\frac{I}{C}\right)}^{-1}.$$

where C is the regularization constant of the ELM-AE. In addition, the initial values of ELM-AE-IW’s input weight, ELM-AE-HW’s input weight and hidden layer’s output $H_{0}$ are assigned by the standard normal distribution.

2. Online sequential learning stage:

In the online sequential learning stage, the weight matrix will be updated once a new set of input data with $N_{k+1}$ training samples arrives. x(k+1) represents the newly added sample here. According to the RLS method, the forgetting factor λ is introduced, then the updated equations of the model are as follows:

(6) $$\beta_{0}=0,\;\;\;\;P_{0}={\left(\frac{I}{C}\right)}^{-1}.$$

The input weight update is $W_{k+1}=\beta_{k+1}^{i}{}^{T}$, where

(7) $$\beta_{k+1}^{i}=\beta_{k}^{i}+P_{k+1}^{i}H_{k+1}^{i}{}^{T}(x(k+1)-H_{k+1}^{i}\beta_{k}^{i});$$

(8) $$P_{k+1}^{i}=\frac{1}{\lambda }P_{k}^{i}-P_{k}^{i}H_{k+1}^{i}{}^{T}{\left(\lambda^{2}+\lambda H_{k+1}^{i}P_{k}^{i}H_{k+1}^{i}{}^{T}\right)}^{-1}H_{k+1}^{i}P_{k}^{i}.$$

The hidden weight update is $V_{k+1}=\beta_{k+1}^{h}{}^{T}$, where

(9) $$\beta_{k+1}^{h}=\beta_{k}^{h}+P_{k+1}^{h}H_{k+1}^{h}{}^{T}(H_{k}-H_{k+1}^{h}\beta_{k}^{h});$$

(10) $$P_{k+1}^{h}=\frac{1}{\lambda }P_{k}^{h}-P_{k}^{h}H_{k+1}^{h}{}^{T}{\left(\lambda^{2}+\lambda H_{k+1}^{h}P_{k}^{h}H_{k+1}^{h}{}^{T}\right)}^{-1}H_{k+1}^{h}P_{k}^{h}.$$

The Output matrix update is

(11) $$H_{k+1}=g(norm(W_{k+1}x(k+1)+V_{k+1}H_{k}))$$

where, *g*() is the activation function.

The output weight update is

(12) $$\beta_{k+1}=\beta_{k}+P_{k+1}H_{k+1}^{T}(x(k+1)-H_{k+1}\beta_{k})$$

(13) $$P_{k+1}=\frac{1}{\lambda }P_{k}-PkH_{k+1}{}^{T}{\left(\lambda^{2}+\lambda H_{k+1}P_{k}H_{k+1}{}^{T}\right)}^{-1}H_{k+1}P_{k}.$$

**Figure 2:**
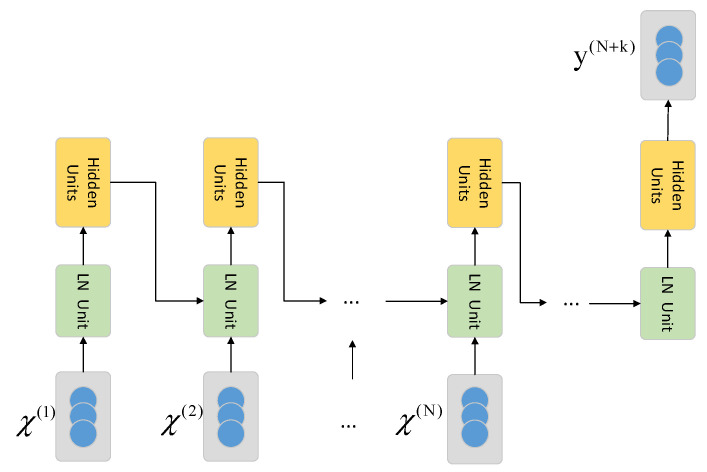
The architecture of the OR-ELM model.

The OR-ELM based prediction network designed in this paper is shown in Figure 2. The input layer of the model consists of N cells, and each cell contains a vector of L dimensions arranged in specific order. The output layer of the model contains only one-dimensional scalar value. The input layer and the output layer are sequentially connected by the LN layer and the hidden layer. The output of a hidden layer’s cell is input to the LN layer of the next cell together with the original data at next time step after the multiplication operation with the specific weight coefficient.

The input of the model consists of a set of time series data $\chi =\{\chi^{\left(1\right)},\chi^{\left(2\right)},\ldots ,\chi^{\left(N\right)}\}$, where *N* represents the length of time window required. The OR-ELM-based prediction model proposed in this paper is only applicable to a certain position’s temperature prediction task. Each element $\chi^{\left(t\right)}\in R^{L}$ in the input sample χ is composed of an m-dimensional vector $\left\{\chi_{1}^{t},\chi_{2}^{t},\ldots ,\chi_{L}^{t}\right\}$, where *L* represents the length of the feature table and the scalar value $\chi_{k}^{t}$ represents the value of the (*k*)th input at time *t*. Here we set the value *L* as 11, then the *L* elements in each $\chi^{\left(t\right)}$ correspond exactly to the *L*-dimensional inputs.

The output of the model is also a set of time series data $Y=\{y^{\left(N+k\right)}\}$, where $y^{\left(N+k\right)}$ represents the predicted temperature after *k* cycles at the measurement point.

### 3.3. Chaotic Ensemble of OR-ELM

**Figure 3:**
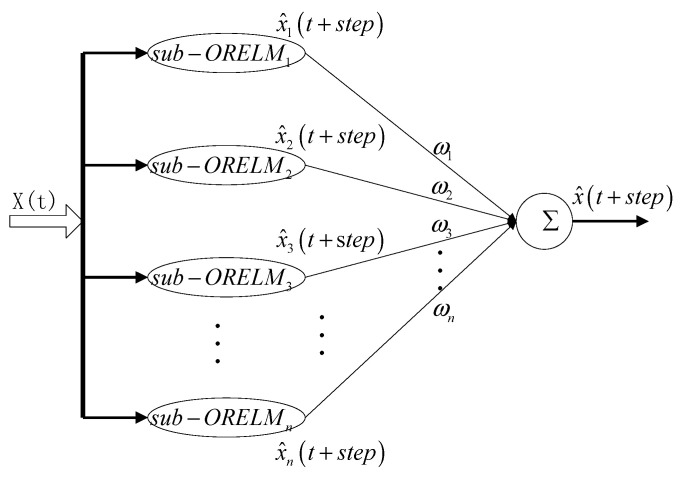
The structure of chaotic ensemble of OR-ELM (CE-ORELM).

The OR-ELM network takes the minimum embedding dimension as the input number. Therefore, the estimation of the minimum embedding dimension has a great influence on the accuracy of the network. However, in practice, it is difficult to get the exact embedding dimension using the GP algorithm, and the stability of a single OR-ELM network is poor, which leads to the inaccurate prediction results of a single OR-ELM network. Therefore, a chaotic ensemble of OR-ELM consisting of multiple OR-ELMs (CE-ORELM) is proposed in this paper. The structure is shown in Figure 3. In this model, multiple parallel connected OR-ELM networks are constructed, which are denoted as $sub-ORELM_{i}$ (*i* = 1, 2, …, *n*). The prediction results of each network are integrated with proper weights to obtain the final prediction results of the network, to improve the prediction accuracy. In this paper, each subnet uses the default parameter, where the number of hidden layers is 1, and the number of hidden nodes is equal to the number of input vectors.

The correlation dimension *D* is obtained by the G–P algorithm described above, and the minimum embedding dimension *m* is determined according to the Formula (2). Let the number of input nodes in the central subnet $sub-ORELM_{\left[n/2\right]}$ be equal to *m*. The number of input nodes in other subnets is defined as follows:

(14) $$In_{i}=In_{\left[n/2\right]}+\left(i-\left[n/2\right]\right)(i=1,2,\cdots ,n),$$

where *n* is the total number of subnets in CE-ORELM. $In_{i}$ represents the number of input nodes in the subnet. When i=[n/2], the subnet is called central subnet.

Due to the instability of the performance of a single ORELM network, each subnet needs to be weighted appropriately to obtain more accurate prediction results. Define the weighted factor as *ω*, the optimal weighted factors of each subnet *ω_i_* (*i* = 1, 2, …, *n*) is calculated through the least square regression algorithm.

(15) $$minJ_{CE-ORELM}=min\sum_{t=1}^{N}{\left[x\left(t+step\right)-\sum_{i=1}^{n}\omega_{i}{\hat{x}}_{i}\left(t+step\right)\right]}^{2},$$

where, *N* is the number of samples, step is the prediction step and x(t+step) is the true value.

The final prediction results $\hat{x}\left(t+step\right)$ of CE-ORELM network can be expressed as follows:

(16) $$\hat{x}\left(t+step\right)=\sum_{i=1}^{n}\varpi_{i}\times {\hat{x}}_{i}\left(t+step\right),$$

where, *n* is the total number of subnets in CE-ORELM and ${\hat{x}}_{i}\left(t+step\right)$ represents the output of the ith subnet.

### 3.4. Framework of Temperature Prediction

**Figure 4:**
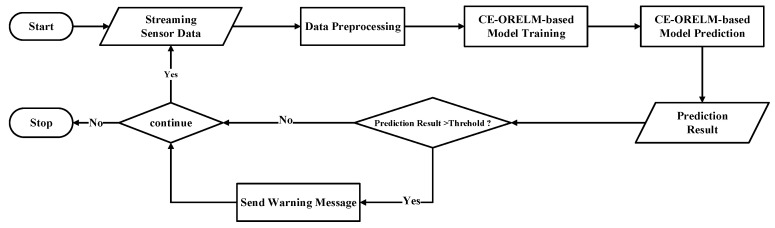
Framework of the prediction model.

As shown in Figure 4, the CE-ORELM-based temperature prediction framework proposed in this paper is mainly composed of three parts: data preprocessing part, CE-ORELM-based model’s training and prediction part, and an auxiliary alarm part.

The CE-ORELM model predicts the temperature data of each measurement point in the next k cycles. In the comparison link, the prediction value was compared with the temperature warning threshold of each measurement point, and the difference between the prediction value and the external environment temperature was compared with the temperature warning threshold of each measurement point. When any one of the prediction values exceeded the threshold, the warning signal would be sent.

## 4. Experiments

### 4.1. Data Description

**Figure 5:**
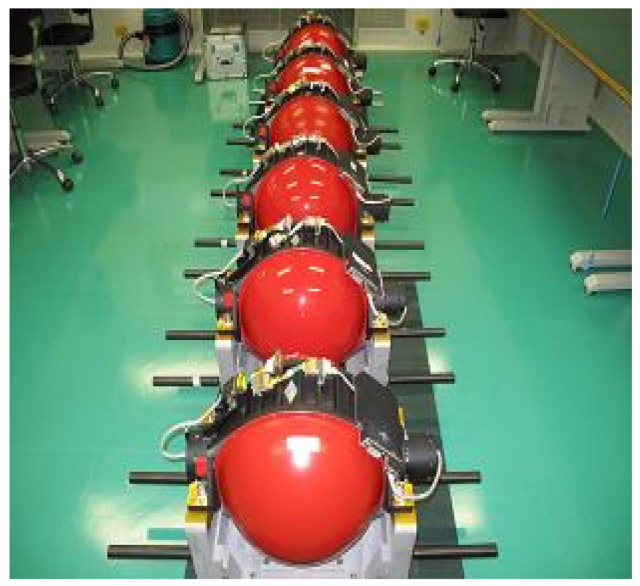
Control moment gyroscopes.

**Figure 6:**
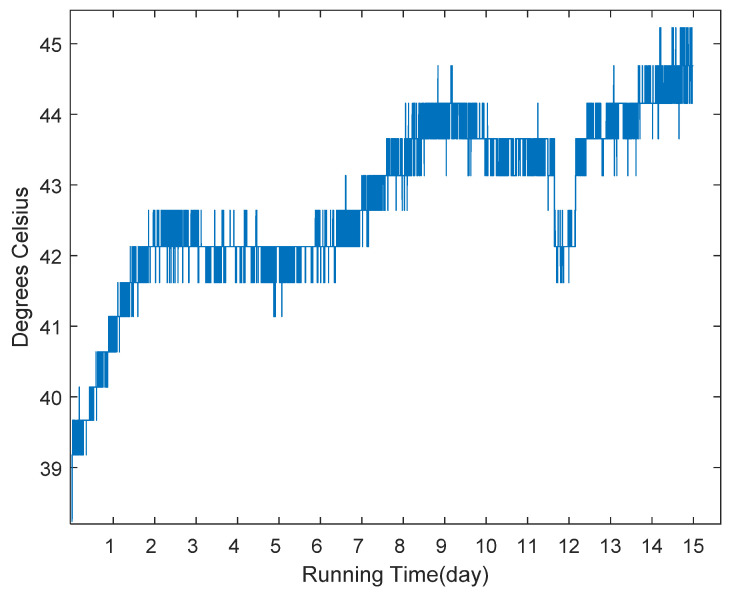
The raw data.

The data used in the experiments are collected from control moment gyroscopes of a satellite in service. The temperature sensor installed on the high-speed bearing of each CMG which is shown in Figure 5. We collected the running data within 15 days and stored them in the offline database, and the raw data is shown in Figure 6. If the temperature value is larger than 70 °C, the CMG is considered to be breaking down. To simulate the online scenario as much as possible, we arranged this data into streaming data format in time order for the use of prediction task.

There are two hyper parameters that need to be determined before applying the model to make predictions tasks. They are Μ and λ, which respectively represent the number of hidden nodes and the forgetting factor of model. Finding the optimal hyper parameters becomes a key task for the data with different distributions. We divided the dataset into three parts $\nu_{n}$, $\nu_{t}$ and $\nu_{m}$. To evaluate the feasibility and adaptability of the model in response to emergencies, we filtered out a piece of data containing special events (emergency braking in our experiment) from the data set, which is $\nu_{m}$. Then we divided the remaining data into $\nu_{n}$ and $\nu_{t}$ according to the ratio of 8:2. The data set $\nu_{n}$ was used to train the optimal hyper parameters of the model, and $\nu_{t}$ was used to evaluate the model’s performance in solving this prediction problem and to compare its characteristics with other models.

### 4.2. Hyper Parametric Setting

To obtain the optimal hyper parameters M and λ, the data set $\nu_{n}$ is involved. According to the structural characteristics of the model itself, the optimal value of the parameter λ is between the interval 0.9–1. We first fix a certain λ, and then test the prediction accuracy of the model obtained after taking different *M* (10–1500) on the data set $\nu_{n}$. Finally, we can get the optimal *M* with this λ. By constantly adjusting the value of λ, the optimal hyper parameters M and λ applicable to the data set can be obtained after some iterative searches.

### 4.3. Experiment Scheme Design

We conducted experiments to compare the performances of the proposed CE-ORELM for temperature prediction with different parameters. To evaluate the performance of the prediction result on the test data set, we introduce the normalized root mean square error (NRMSE) and mean absolute percentage error (MAPE) indicators for analysis. In the time series prediction, NRMSE is the most common evaluation indicator used to evaluate the performance, which can reflect the similarity between the prediction series and the actual series. It can be calculated as:

(17) $$\mathrm{NRMSE}=\frac{\sqrt{\frac{1}{T}\sum_{k=1}^{T}{\left(Y_{k}-y(k)\right)}^{2}}}{Y_{\mathrm{meam}}},$$

where *T* represents the length of data sequence, $Y_{k}$ represents the observed value at time *k*, and *y*(*k*) represents the predicted value of the algorithm at time *k*.

Although data preprocessing methods were implemented, there were still some noises contained in data. Contrary to NRMSE, MAPE is more suitable to evaluate the performance of prediction models with noisy data. It can be calculated as:

(18) $$\mathrm{MAPE}=\frac{\sum_{k=1}^{T}\left|Y_{k}-y(k)\right|}{\sum_{k=1}^{T}\left|Y_{k}\right|},$$

where *T*, $Y_{k}$, and *y*(*k*) are defined the same as NRMSE.

### 4.4. Parameters of CE-ORELM

The correlation dimension *D* can be obtained through the G–P method described above. Firstly, a cluster of ln *C_m_*(*r*)–ln(*r*) curves of the normal data are plotted through increasing *m*. Then the least square regression method is used to obtain the estimated value of *D*. According to the Formula (2), the embedding dimension *m* is determined, and *m* = 6. After training and testing, a prediction model of normal state can be determined.

## 5. Results and Discussion

**Figure 7:**
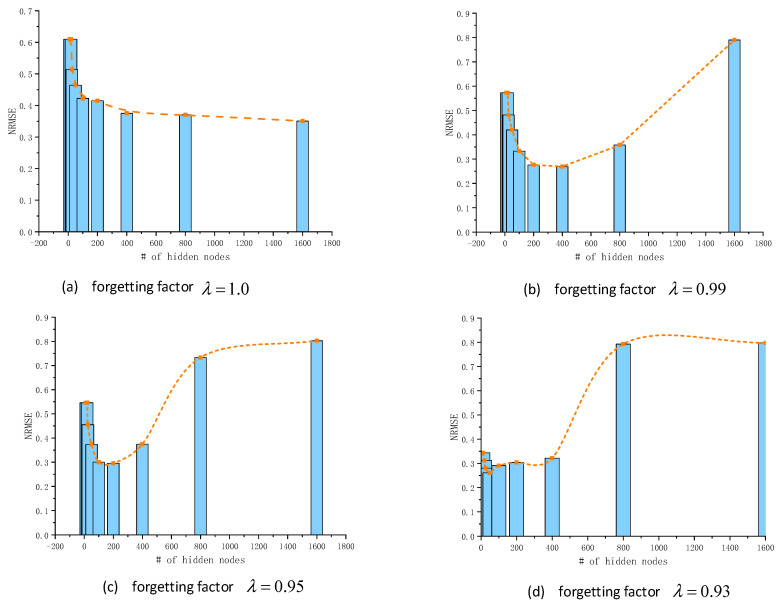
Performance on the prediction by Chaotic Ensemble of Online Recurrent Extreme Learning Machine (CE-ORELM).

The experimental results are illustrated in this section. By analyzing the prediction error of the model with different forgetting factors λ and hidden node number *M*, as shown in Figure 7, we obtained the optimal hyper parameters in this experiment.

The algorithm proposed in this paper is verified by the test data $\nu_{t}$ mentioned above. At the same time, the performance of the proposed CE-ORELM algorithm is compared by the NRMSE and MAPE in different training samples and prediction steps. The results are shown in Table 1. In terms of training sample 3000, the NRMSE values are 0.0021, 0.015, 0.0028 and 0.0030 respectively, which are lowest in all the NRMSE values, including the training sample 2500 (0.0042, 0.1400, 0.0224 and 0.1423) and the training sample 2000 (0.0118, 0.1179, 0.0423 and 0.0466). Therefore, the number of the training sample has certain influences on the performance of CE-ORELM. The MAPE are 0.0014, 0.0012, 0.0025 and 0.0031 respectively, which is lower than other training samples (2500: 0.0107, 0.0226, 0.0165 and 0.0340; 2000: 0.2250, 0.5130, 0.2515 and 0.3659). Therefore, the CE-ORELM algorithm is robust and effective in the forecasting of time series with intrinsic nonlinearity and chaotic characteristics.

**Figure 8:**
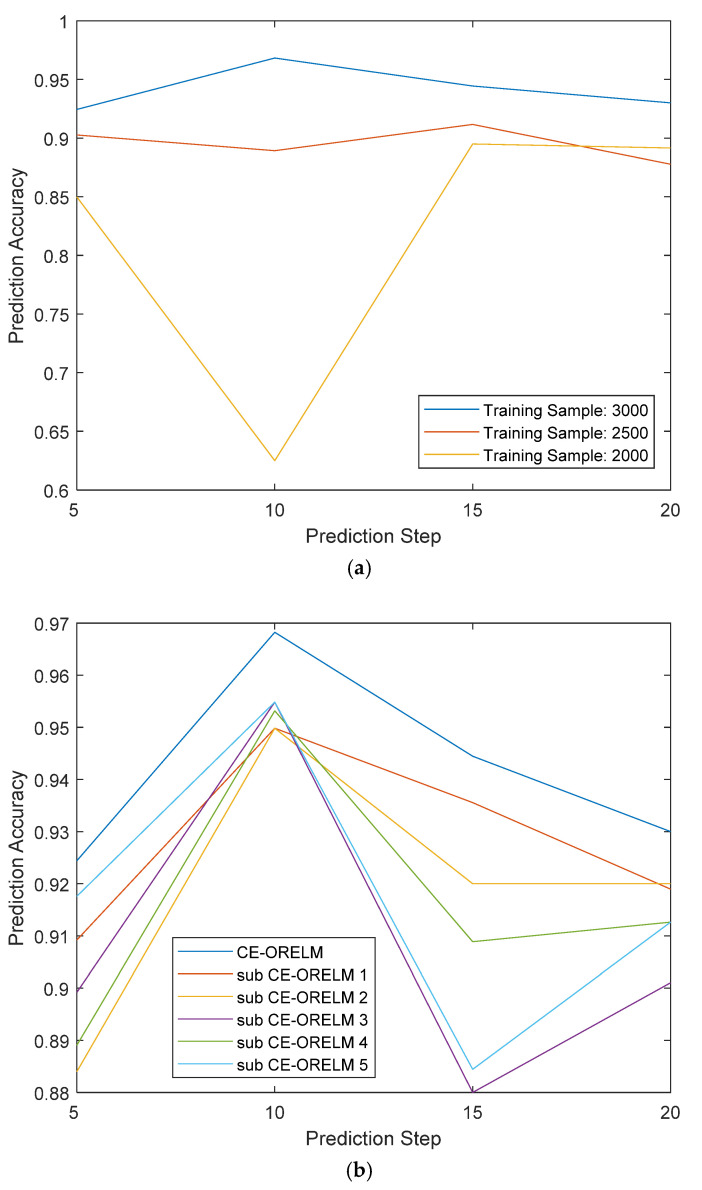
Prediction accuracies (**a**) under different samples (**b**) under different sub CE-ORELM.

In order to compare the performance of the models with different prediction steps and training data, we selected different prediction steps of 5, 10, 15 and 20, and training samples of 2000, 2500 and 3000 to analyze the prediction accuracy respectively, as shown in Figure 8. It is shown in Figure 8a that when the number of training samples is 3000, the prediction accuracies of all models perform best compared with the training samples of 2500 and 2000 and are all above 90%. Meanwhile, the accuracies of training samples 2500 are around 90%, and the accuracies of training samples 2000 are from 60% to 90%. Therefore, it can be seen from Figure 8a that with the number of training sample increases, the accuracy of CE-ORELM can reach about 90% gradually. The greater the number of training samples, the higher accuracy it achieves. According to Figure 8b, with the different prediction steps, CE-ORELM has the highest accuracies all the time (no less than 92%), compared with the sub-ORELMs. What is more, as shown in both Figure 8a,b, the prediction accuracies of prediction step 10 is almost the best with different parameters. Therefore, the proposed algorithm with the prediction step of 10 can be used for temperature prediction of control moment gyroscopes.

**Figure 9:**
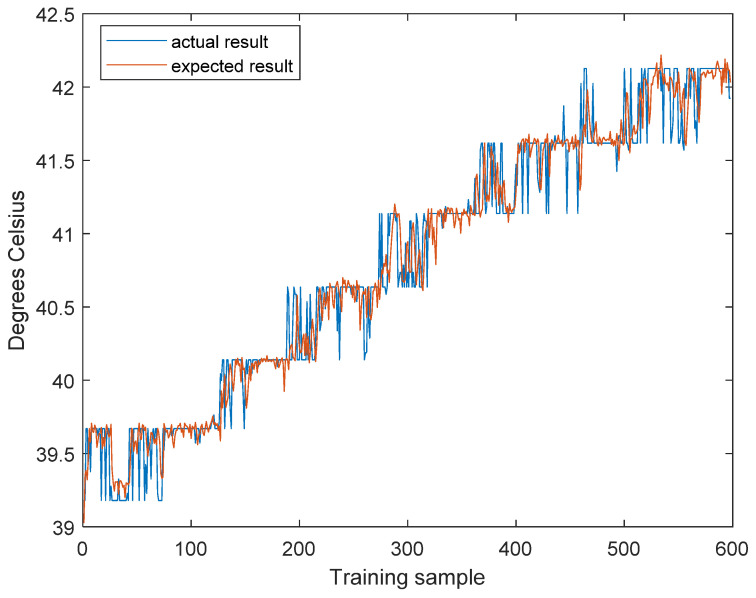
Prediction result.

To demonstrate the efficiency of the CE-ORELM in details, a number of results are shown in Table 2 and Figure 9. The weights of sub-ORELMs with the prediction step 10 are shown in Table 2, and the actual outputs and predicted outputs with prediction step 10 are shown in Figure 9. It is obvious that the curve of actual outputs and the curve of predicted outputs are almost the same. Moreover, the predicted outputs (ranging from 39 to 42) are not only close to actual data, but also indicate the same variation trend with the actual data, which has obvious variable stages. Therefore, the proposed method is effective in temperature prediction under different operation states.

Moreover, as shown in Figure 9, it is noticeable that due to the regular change of thermal environment on orbit, the temperature of the high-speed bearing increases with step-wise changes and the values fluctuate between adjacent stages. In this scenario, the proposed method tends to get close to the later stage. Although this tendency decreases the accuracy, it is helpful for us to decide ahead of time. It is also shown that when the suddenly varying data emerges, the proposed CE-ORELM can also perform well. Therefore, it is obvious that the CE-ORELM has high prediction accuracy and strong adaptability to the on-orbital temperature data with sudden variations.

## 6. Conclusions

Temperature prediction is a significant part of the health management system for CMGs. This paper proposes a chaotic ensemble-ORELM-based framework that can be applied to the temperature prediction of CMGs. We tested this framework with the actual data acquired in the running process of CMGs. The experiment results show that the prediction accuracy increases with the increase of the number of training samples. The prediction step also has influence on the prediction accuracy, and the accuracy is highest with the prediction step 10 in the experiment. The experimental results show that this framework outperforms others with a higher prediction accuracy over 96%. What is more, the proposed framework has a good ability in predicting the data with sudden variations and is therefore effective in temperature prediction under different operation states. These advantages indicate that this framework approximately meets the requirements of temperature prediction of CMGs in practice.

However, the high accuracy is based on the sufficiency of training samples. In practice, the absence of fault data may create over-fitting. Thus, more experiments with small sample data and imbalanced data should be done in the future.

## Figures and Tables

**Table 1:** The analysis of prediction results based on normalized root mean square error (NRMSE) and mean absolute percentage error (MAPE).

Training Sample	Value	Prediction Step 5	Prediction Step 10	Prediction Step 15	Prediction Step 20
3000	NRMSE	0.0021	0.0015	0.0028	0.0030
	MAPE	0.0014	0.0012	0.0025	0.0031
2500	NRMSE	0.0042	0.1400	0.0224	0.1423
	MAPE	0.0107	0.0226	0.0165	0.0340
2000	NRMSE	0.0118	0.1179	0.0423	0.0466
	MAPE	0.2250	0.5130	0.2515	0.3659

**Table 2:** Weights of sub-online recurrent extreme learning machine (ORELMs).

Sub-ORELM	Embedding Dimension 4	Embedding Dimension 5	Embedding Dimension 6	Embedding Dimension 7	Embedding Dimension 8
Weight	0.3454	0.1350	0.1522	0.2358	0.1316
